# Organizational-level determinants of participation in workplace health promotion programs: a cross-company study

**DOI:** 10.1186/s12889-019-6578-7

**Published:** 2019-03-06

**Authors:** Liesa Marie Lier, Christoph Breuer, Sören Dallmeyer

**Affiliations:** Institute of Sport Economics and Sport Management, German Sport University Cologne, Cologne, Germany

**Keywords:** Workplace health promotion, Employee participation, Organization level determinants, Implementation effectiveness, Physical activity

## Abstract

**Background:**

Attracted by the expected benefits such as reduced absenteeism and increased productivity, more and more firms decide to implement workplace health promotion programs (WHPPs). However, those programs can only be effective if employees actually participate. This study aims to (1) gain insight into the degree of enrolment rates in such programs across companies and (2) identify organizational level factors that are associated with employee participation. Building on existing theory on organizational drivers of participation in corporate wellness programs, the study’s main goal is to investigate which organizational factors determine whether employees enroll in a corporate fitness program or not.

**Methods:**

A business-to-business fitness platform company provided organizational level data on 61 client firms that have recently implemented a corporate wellness program. The data contained information on the enrolment rate per company and different organizational level variables. The following potential determinants of participation were analyzed: firm size, organizational program support and employee co-payment. A random effects model was used to examine associations between potential determinants and the program enrolment rate.

**Results:**

The average participation is limited (15.37%) but varies highly across companies (range 0.07–100.00%, monthly basis). Looking at the determinants of program enrolment, we find that organizational program support – the degree to which firm leadership encourages participation – positively influences the enrolment rate (β = 0.051 *p* < 0.001) while employee co-payment – the financial contribution employees have to make to participate – has a negative impact (β = − 0.002, *p* < 0.001). Furthermore, firm size has a negative relationship with firm enrolment.

**Conclusions:**

Enrolment rates in WHPPs are limited, as many companies have difficulties to promote participation in WHPPs among employees. Strong organizational program support and low employee co-payment were identified as drivers of employee participation in corporate health programs. Hence, intensifying both social and financial support of employee participation may help to drive enrolment rates. Firm size was found to negatively affect the enrolment rate in WHPPs, implying that larger firms have to account for their size and corresponding complexity when implementing such a program.

## Background

Despite the plentiful evidence of the positive effects of physical activity on both physical health [1, 2] and general well-being [3–5], a significant share of the world’s population is not active enough [6]. In an attempt to reach a high share of the population, public health authorities encourage employers to promote physical activity at the workplace, where most adults spend a significant amount of time [7–10].

However, even independent of any external encouragement, firms may be inherently interested in increasing physical activity levels among their workforce, as they occur indirect costs of physical inactivity stemming from productivity losses related to increased absenteeism and a lack of productivity at work [11]. Several researchers have conducted studies in the field of labor market outcomes of physical activity – proving that physical activity reduces employee absenteeism [12–15], health care costs [16, 17] and improves employee-performance at work [11, 18].

Given the evidence on the employer benefits of health promotion among employees, it is no wonder, that more and more companies introduce corporate health programs or have plans to do so [19, 20]. However, any program can only be effective, if employees actually participate. Unfortunately, current participation rates remain far below expectations [19, 21]. These low participation rates severely limit the effectiveness of physical activity promotion programs, in particular if they occur among high-risk groups.

To address the issue of low employee participation rates and design programs that achieve higher ones, it is essential to detect the most influential factors of participation. What are the factors that distinguish programs with high participation rates from those with low ones? Different social ecological models on health promotion [22–24] propose that factors on multiple levels play a role in influencing an employee’s decision to participate in a corporate wellness program or not. McLeroy et al. [23] distinguish between – among others – intrapersonal, interpersonal, and organizational factors that affect the outcome of health promotion programs. Intrapersonal factors center on the single individual, interpersonal factors focus on interaction patterns within small groups, and organizational factors relate to the social norms and the corporate climate prevalent at the worksite as a whole [25] p594. The impact of individual level factors on physical activity behavior has been widely researched [26–31]. Employers certainly can (and should) consider those individual level factors in order to drive program participation when designing a fitness program, but they have very limited room to exert influence. Organizational level factors on the other hand are in the firm’s control – however, there is a clear lack of studies on organizational-level variables as predictors of program participation.

While empirical studies on the relationship between organizational-factors and participation are scarce, there does exist some scientific literature. Several researchers who looked into the association between organizational level factors and participation suggest that manager and co-worker support has a positive effect on employee program participation [32–34]. The degree to which employees feel that a certain behavior is socially expected and encouraged at the workplace is predicted to have an impact on program participation [35]. However, the cited studies only developed theoretical hypotheses on the association between social support and participation and did not test it empirically. Nonetheless, some qualitative studies on corporate-health-promotion success factors confirmed that organizational program support is of great importance [36, 37]. Another factor that receives particular attention is the usage of (financial) incentives to motivate participation [12, 21, 38]. Given the growing evidence of the positive effect of financial incentives on behavior change [39–42], it is no wonder that this topic receives great attention. Lastly, firm size is mostly hypothesized to have a negative influence on participation in corporate health programs [43, 44], as the closer connections among employees prevalent at small workplaces encourage participation in a health promotion program [45] p331.

As both industry participants and researchers have not yet arrived at an understanding of the determinants of – currently limited – employee participation in corporate health programs, there is a clear need to investigate this topic. This study aims to (1) gain insight into the degree of enrolment rates in such programs across companies and (2) identify organizational level factors that are associated with employee participation. This research is highly relevant for companies that consider implementing a WHPP as it provides insights on how to design such a program in a way that maximizes participation levels. Furthermore, it contributes to the advancement of research, as we are the first to analyze data of a fitness platform company that is interacting as intermediary between members and a variety of fitness outlets – a business model that is currently disrupting the fitness industry.

## Method

### Data sources and sample description

To test the impact of organizational level variables on employee participation in corporate health programs, we collaborated with a corporate fitness platform (‘Platform A’) that offers corporate clients access to a flexible physical activity and wellness program. By supervising the process of data extraction and an immediate handover, we assured completeness of the data provided. Building on prior research, the authors suggested a study design which was presented to and confirmed by Platform A employees who supported the results interpretation. The data set comprises organizational level information on all Platform A clients in Germany since the national market entrance in early 2017. The time period covered is January 2017 until August 2018 (20 months) as this was the latest recording available when we conducted the study. The total sample comprises 65 companies. Four high-priority customers who received special promotional offers have been excluded due to a lack of comparability. Consequentially, the final sample consists of 61 companies. It is important to mention that the number of data points available per company varies for the data that is reported monthly, as they started working with Platform A at different points in time. Depending on the length of collaboration between the clients and Platform A, there is data available for between 2 and 20 months, with an average of 9 months. Overall, the dataset includes *n* = 550 observations. The client companies operate in six different industry groups (Tech & Media, Services, Retail, Public Administration, Industrial and Hotel) and have been classified as white collar (43 companies) or blue collar (18 companies) employers, depending on what type of work the majority of their employees engage in. As the data set comprises only company level data and does not include any employee level data, an ethics approval was not required.

### Measures and variables

Empirically determining those organizational factors that influence the likeliness of achieving high participation in corporate wellness programs is the main purpose of this paper. Hence, our dependent variables is a measure of participation, the enrolment rate. The enrolment rate is calculated by dividing the total number of employees who are enrolled by those who are eligible for program enrolment. Some clients decided to launch the program gradually at different company locations; hence, the denominator may vary over time. The enrolment rate is reported on a per-month basis.

Five organizational level variables have been reported: industry, type of work (blue collar vs. white collar), organizational program support, employee co-payment and firm size. After reviewing existing literature, we expect the last three of those variables – organizational program support, co-payment and firm size – to influence program participation. Hence, we included those as predictor variables in our model while the other two serve as control variables. *Organizational program support* summarizes whether the fitness program receives support within the client organization. A team of Platform A employees responsible for promoting program participation (the engagement team) assigned an ‘organizational support score’ for each company. They assessed to which degree the leadership team of the respective client supports the corporate fitness program and promotes its success. When conducting the assessment, the engagement team looked at several factors that can be categorized into a) willingness to allow Platform A to conduct activation and engagement activities (passive support) and b) effort to promote the program within the company (active support). Passive support comprises elements such as whether the client firm management has allowed Platform A to contact employees directly for motivational purposes or to conduct activation events on company premises. Active support refers to the general attitude of the management towards fitness and health topics. Questions such as whether the firm has made health a priority and acts accordingly (e.g. leadership encourages health behavior, leadership participates in program and serves as role model) are answered in this context. Both variables are assessed on a scale from zero (=very low) to ten (=very high) and averaged to determine the final variable score. Organizational program support is hence an ordinal variable.

*Employee co-paymen*t is the net Euro amount each enrolled employee pays to participate in the fitness program. The costs of the program are shared between employer and employees. Hence, the employee co-payment varies, depending on how much the employer is willing to cover: it decreases as the investment made by the firm increases. There is no employee level data available but only information on the total employee co-payment, which is then divided by the total number of employees; thus, we take a per-employee average for this variable. Co-payment is reported on a per-month basis.

*Firm size* is measured by the total number of employees working at the client organization at the point of contract conclusion. While the companies may have been growing since then, we only focus on the point-in-time number of employees as we argue that the organizational size at the start of the partnership has an impact on how well the new initiative is adopted companywide. For the analysis of the effect of size, we transformed the continuous variable in a categorical one: Firms with 0–50 employees, 51–200 employees, 201–500 employees and > 500 employees. We decided to categorize this predictor as it enables us to uncover a potential relation between firm size and enrolment rate without requiring the values of the predictor to follow a line or fitting a complex non-linear model.

### Data analysis

Three independent variables of interest on the firm level were considered (co-payment, program support, firm size), which are hypothesized to have a linear relationship (the direction of the relationship depends on the specific variable pair) with the enrolment rate. In addition, we included control variables for firm industry and type of work. As the data contains repeated observations of firms on a monthly basis, we decided to apply a random effects model to account for the individual effects of the different observations and potential correlations among repeated measurements, as well as for inter-company differences that are unexplained by the predictors. The standard errors are clustered at the firm level to consider the fact that the same firm is observed over several months. All statistical analysis have been carried out with the program Stata.

The following model equation was formulated to analyze the relationship between the potential determinants and the enrolment rate:

$$ {Yenrolment}_{it}=\mathrm{b}0+\mathrm{b}1{\left( co- pay\right)}_{it}+\mathrm{b}2{\left( program\ support\right)}_i+\mathrm{b}3{\left( firm\ size\right)}_i+\mathrm{b}4{\left( work\ type\right)}_i+\mathrm{b}5{(industry)}_i $$

where *Yenrolment*_*it*_ captures the average enrolment rate of firm i in month t and the three independent variables organizational program support, employee co-pay and firm size are said to predict enrolment. The only independent variable that varies monthly is co-pay. The independent variables under consideration might be correlated which has to be accounted for when interpreting the results. Hence, to assess the existence of any collinearity among the independent variables, variance inflation factors (VIFs) were calculated. As the data set only contains organizational variables, the model is not suitable to predict all variance in the enrolment rate. The enrolment rate also depends on individual level factors such as gender, age, base health factors and many more.

## Results

Tables 1-2 summarize the descriptive statistics and regression results of the analysis. The data set includes *n* = 550 observations from *n* = 61 firms with between 3 and 16,000 employees. The average enrolment rate in the corporate health program is 15.37% (SD: 20.59%) and the average co-payment in firms is 18.62€ (SD: 10.18€). The program support is on average at 4.01 (SD: 1.79). Regarding firm size, 18.18% of the firms have between 0 and 50 employees, 42.0% have between 51 and 200 employees, 19.46% between 201 and 500, and 20.36% over 500 employees.

**Table 1:** Means, Standard Deviations and ranges of key measures

Measure	*n*	*M*	*SD*	*Minimum*	*Maximum*
Enrolment	550	15.37	20.59	0.70	100.00
Co-pay	550	18.62	10.18	0.00	59.24
Program support	550	4.01	1.79	1.00	8.50
Firm size 0_50	550	0.18		0.00	1.00
Firm size 51_200	550	0.42		0.00	1.00
Firm size 201_500	550	0.20		0.00	1.00
Firm size > 501	550	0.20		0.00	1.00
White collar	550	0.74		0.00	1.00
Blue collar	550	0.26		0.00	1.00
Tech. media	550	0.31		0.00	1.00
Hotel	550	0.12		0.00	1.00
Industrial	550	0.11		0.00	1.00
Public admin.	550	0.11		0.00	1.00
Retail	550	0.05		0.00	1.00
Services	550	0.31		0.00	1.00

**Table 2:** Predicting enrolment from co-pay, program support and firm size

	*β*	SE	*z*	*P* > |*z*|	VIF
*Firm variables*					
Co-pay	−0.002	0.000	−3.50	0.000	3.87
Program support	0.051	0.014	3.71	0.000	3.54
Firm size 51_200	−0.188	0.070	−2.68	0.007	3.30
Firm size 201_500	−0.203	0.061	−3.33	0.001	2.02
Firm size > 501	−0.253	0.067	−3.80	0.000	2.02
*Control variables*					
Blue collar	−0.100	0.060	−1.67	0.094	4.99
Hotel	0.380	0.066	0.57	0.567	3.02
Industrial	0.116	0.100	1.16	0.245	3.37
Public admin.	0.143	0.092	1.56	0.120	1.35
Retail	0.035	0.680	0.52	0.001	1.49
Services	0.012	0.044	0.29	0.000	1.96
Constant	0.145	0.078	1.86	0.063	
Num. Observations			550		
Num. Firms			61		
Wald-Chi			76.23(p < 0.001)		

*Note:* Model fit: R^2^ within =0.049, R^2^ between = 0.593, R^2^ total = 0.609; Reference categories are: Firm size 0_50 (Firm size), Blue collar (Work type), and Tech. Media (Industry). Clustered standard errors are applied

When the relationship between the variables and enrolment was investigated, it was found that organizational program support (β = 0.051, *p* < 0.001) and co-payment (β = − 0.002, p < 0.001) are significant predictors for program enrolment. Also, firm size has a significant effect on enrolment: the enrolment rate is highest for companies in the 0–50-employees category and gradually decreases as the number of employees increases for the other three categories (Firm size 51–200: β = − 0.188, *p* < 0.01; Firm size 201–500: β = − 0.203, *p* < 0.01; Firm size > 500: β = − 0.253, *p* < 0.001). The overall model fit was R^2^ = 0.609, hence the model containing organizational program, employee co-payment, and size can explain 60.91% of the variation in enrolment). The collinearity statistics show that we do not face the problem of multicollinearity in this study (variance inflation factors < 10) [46].

## Discussion

Primary purpose of this paper was to bring clarity into the matter of designing a corporate health program that achieves high participation rates. Building on prior literature, we identified three potential determinants and empirically analyzed their impact on participation, using data from practice. The results confirm and find significant support for the positive impact of both a supportive organizational environment and low employee co-payments. Given the fact that we only considered a subset of organizational- and program level factors and omitted individual level factors altogether, the model can be considered a good fit for the data set at hand, as it explains more than 60% (R^2^ = 0.609) of the variation in enrolment. We did not only assess the impact of those factors that can be actively adjusted by the shapers of a corporate health program – the firm itself and the fitness platform provider –, but also tested for a potential structural disadvantage of large firms. It was indeed revealed that firm size in terms of employees has a negative relationship with employee enrolment in the WHPP.

Generally, when interpreting the results one should keep in mind that the data set contains a relatively small sample of only 61, which certainly limits the explanatory power of this study. Nonetheless, with our findings, we advance the understanding of determinants of participation rates in corporate health programs as we empirically analyze the impact of organizational level factors on enrolment with a sample of companies of from different industries. This study is – to our knowledge – among the first to investigate the specifics of the new platform fitness model, which becomes more and more popular among firms that consider implementing a corporate health program. Our results show that advancing the understanding of the success factors of modern corporate health programs is critical: The average enrolment rate of the analyzed program is only around 15%. In order for an organizational health program to have positive effects, employees have to enroll and actively use the program. 15% enrolment is clearly below the average participation rates of 20–30% prior research has reported [21], which is unexpected. A potential reason might be that the way of engaging with a fitness aggregator platform is still in its infancy: Collaboration models between aggregator and client still have to be further developed; roles and responsibilities (e.g. ongoing employee engagement) have to be defined. Corporate wellness programs are much more common in North America, where 52% of employed workers have access to such a program, than in Europe (23%) [47]. Hence, the German employees might have to get used to those programs. A second factor that should be taken into account when evaluating this enrolment rate is that the German employees are generally more concerned with the privacy of any data related to their health than are citizens of other countries [48] – hence, they might be less interested in worksite health promotion programs. While these two arguments may explain enrolment rate gap partly, they do not change the fact that the observed levels of participation are unsatisfactory. Hence, it can conclude there is still work to do in order to improve the effectiveness of corporate health programs. Our research seeks to support the development of an understanding of several factors that may affect participation in those programs.

Prior research has already hinted at the high importance of social support: Rongen et al. [49] conducted a study on barriers for facilitation in health promotion programs and found out employees who think that their supervisor or colleagues expect and supports their program participation are more likely to participate. Our results confirm that the degree to which a leadership promotes and facilitates the participation in a corporate health program has an influence on its success. Depending on managerial attitude, this finding can be both blessing and curse. It is fortunate, that a good share of the power to make a corporate health program successful lies within the hands of firm management. However, this power comes with responsibility: It is not possible for a firm to outsource the matter of corporate health completely to partners such as Platform A. While it is helpful to find a partner that actively promotes enrolment in their corporate health program, the preconditions for program success have to be created internally. Establishing a corporate culture in which health is considered a priority is hence not ‘nice to have’ but essential in order to benefit from any corporate wellness program. Saying this, it is striking that the 61 companies in our sample only achieve an average perceived level of program support of 4.01 points – out of 10. The lack of leadership willingness to both actively and passively support organizational fitness programs is concerning and surprising, as a firm’s leadership should have an intrinsic economic interest to boost participation.

Our analysis of the impact of employee co-payment provides support for the idea of employing incentives to drive employee participation in health programs. Prior research has already shown that the provision of financial rewards drives employee participation in health programs [39–42]. In this case, the incentive came in form of a partial reimbursement of the monthly participation fee employees have to pay. Hence, one has to keep in mind that it is different in nature from a simple financial reward that is paid out, as participants still have to cover a share of the cost themselves. This type of incentive is uncritical from a legal perspective, as it is participation- and not success-based. This is important to point out: there is an ongoing ethical debate on the appropriateness of those success-based incentives, as policy makers and researchers worry that they might discriminate against employees with poor health [50–53]. Hence, analyzing alternative forms of financial incentives – such as partial reimbursement – is of practical relevance. The results of this study confirmed a negative linear relationship between co-payment and participation, indicating that the more the employer reimburses, the higher the participation. However, the reimbursement costs not only drive participation and hence potential economic benefits of the program, but also pose a cost factor that directly affects the return on investment of such programs. Hence, it is necessary to keep this trade-off in mind when designing programs. Further research on the ideal amount of reimbursement as part of a monetary cost-benefit analysis of corporate wellness programs is needed before clear guidance can be given.

There is no unequivocal evidence in existing research on the impact of firm size on participation: While some researchers argue for a positive impact of size as larger organization have more resources to push new projects [54], others argue that more complex structures prevalent in large firms lower their ability to implement change effectively [55]. This study indicates that firm size is a significant predictor of enrolment – the larger a company in terms of number of employees, the lower the share of employees who participate in a WHPP. We strongly encourage large companies to keep in mind their size and the associated complexity when implementing a program in order to ensure that all employees throughout the organization have all the necessary information. The heightened organizational requirements and communication difficulties which are often prevalent at larger companies should not been underestimated [45].

In summary, the results pronounce that a leadership team that not only encourages program participation through role model behavior but also establishes a corporate culture in which health is of high value is a key factor for program success. Corporate health programs that are just implemented to ‘Check the box’ of investing in corporate health management are set up for failure. Our findings shall not deter any company from implementing a corporate health program due to the lack of a health culture, but – rather the opposite – seek to encourage firms to realize that physical activity and related health behaviors are of such high importance that they deserve and need a high degree of top leadership attention. A second key learning for firms that are about to implement a health and fitness program is to consider providing thought-through financial incentives for program participation, as our results pronounce that enrolment increases with the amount of employer reimbursement of the program fee. Lastly, firms have to account for their size and potentially associated complexity when planning and conducting the implementation of a firm-wide corporate health program. If they fail to do so, a lagging enrolment rate among employees is to be expected.

### Limitations

Although our entire work is grounded on a valuable data set, which comprises real life information of 61 actively operating companies from various industries, there are several limitations to be discussed. First, one has to consider potential biases of this study when interpreting the results. Information bias, selection bias and the risk of confounding are major concerns that one has to keep in mind. Due to the lack of an accurate, objective measurement of program support, the authors had to draw on a subjective assessment method. As the assessment of ‘organizational program support’ has been conducted by Platform A-employees and is no objective measurement, we clearly face the issue of information bias. One cannot be sure that organizational program support was measured accurately; hence, the validity of the study results may be limited. The fact that another employee who assesses the situation differently might allocate different scores also limits the reliability of this work. Furthermore, selection bias has to be discussed as one could argue that the companies who decided to collaborate with Platform A may share certain characteristics that make them different from other firms. Although the companies in this data set differ in many structural variables, the possibility that commonalities exist cannot be ruled out. Furthermore and despite of the inclusion of control variables, we still clearly face the risk of confounding, as there may be additional factors that we are not aware of which confound the association between the three independent variables and the participation rate. Besides the risk of bias and confounding, there are three other limitations that one has to consider: First, given the limited previous evidence on the impact of organizational- and program-level factors on participation in corporate wellness programs, our results are to a large extent explorative. Numerous additional influencing factors as well as further specific situations could be investigated within our research topic and we pave the way for future research to do so. Secondly, we want to point out the limited generalizability of our results, which is due to our purely German sample. Analyses with samples from other countries or optimally an international data set will help to test the applicability of the results to corporate health programs in other nations. Apart from the ‘German only’ shortcoming, our sample has the additional disadvantage that the data set only comprises 61 companies, as the start-up company Platform A has only been operating for less than two years. Lastly, the results prove that financial incentives drive the enrolment rate; however, we cannot make any statements on the ideal incentive type of incentive or money amount. The authors have analyzed the above-mentioned relationship in isolation while incentives also present a cost factor that negatively affects the return on investment in physical activity programs. Hence, researchers are encouraged to assess the impact of incentives within a full cost-benefit analysis to get clarity on this aspect of program design.

## Conclusion

Using a German data set from Platform A, a corporate fitness platform aggregator for corporate clients, we advance the understanding of organizational and program level factors that influence employee participation in a corporate health program offered by a platform provider. Participation of employees in the program was very limited. Our results pronounce that organizational program support is the key driver behind enrolment, as companies whose leadership encourages participation in health activities see higher participation rates. Furthermore, financial incentives do indeed positively affect enrolment rates in corporate health programs while size has a negative relationship with participation, implying that larger companies have to make a particular effort when implementing a WHPP. This study contributes to research about factors that drive participation in corporate health programs and accentuates the relevance for future work to explore the topic of financial incentives in detail.

## Abbreviation

WHPP: Workplace health promotion program
